# Frontal alpha asymmetry responses to odors linked to burnout severity

**DOI:** 10.1192/j.eurpsy.2026.10922

**Published:** 2026-07-31

**Authors:** S. Tukaiev, J. M. A. Ferreira, M. Makarchuk, I. Zyma

**Affiliations:** 1 Educational Scientific Institute of High Technologies, Taras Shevchenko National University of Kyiv, Kyiv, Ukraine; 2 BrainPatch, Cambridge, United Kingdom; 3 Faculdade de Medicina, Universidade de Coimbra, Coimbra, Portugal; 4 Institute of Biology and Medicine, Taras Shevchenko National University of Kyiv, Kyiv, Ukraine

## Abstract

**Introduction:**

Exposure to odors with different emotional valences activates key brain regions involved in emotion. The emotional impact of odors is modulated by factors such as personality, current emotional state, and attentional focus. Distinct alterations in brain regions involved in emotion processing and regulation are caused by burnout. Frontal alpha asymmetry (FAA), as a well-established electrophysiological marker for emotional processing, has been extensively studied as a potential biomarker for depression and stress, but findings across the literature are mixed.

**Objectives:**

We aimed to investigate the impact of burnout on the odor-induced neurodynamic responses.

**Methods:**

142 participants (female and male) aged 18 to 24 years with no documented manifestations of rhinal pathologies (Taras Shevchenko National University of Kyiv students) were recruited. EEG was registered during the rest state (3 min) and under passive odor stimulation (10 min) with sandalwood essential oil (SEO) (n=37) and izoamilacetate (IA) (n=54). The control group included 51 participants. We estimated the spectral power density (SPD) of all frequencies from 0.2-45 Hz. Frontal EEG asymmetry (asymmetry index) was calculated using subtracting the natural logarithm of spectral power density of alpha band of the left-side channels from the right-side ones (L-R). The 84-item Boyko’s Syndrome of Emotional Burnout Inventory was used detecting the severity of emotional burnout before the registration of EEG.

**Results:**

The Resistance stage of burnout was detected in 23 (control group), 16 (izoamilacetate group) and 13 (sandalwood group) volunteers. We demonstrated that odor-specific olfactory-evoked neurodynamics depending on the burnout severity. Sandalwood essential oil and izoamilacetate were perceived as weakly activating stimuli by all participants and more pleasant by the participants with an unformed resistance stage. A higher left frontal activity under SEO was observed at the first minute of odor presentation while IE leads to a higher right frontal activity. Formation of the Resistence stage of burnout was characterized by a higher left frontal activity during the second minute of exposition to both odors.

**Conclusions:**

Under non-directed attention conditions of passive odor perception, topographical reorganization of brain activation reflects information and emotion-activating processes. We detected that unique odor-induced responses are influenced by the formation of burnout.

**Disclosure of Interest:**

None Declared

